# Metformin reduces HGF-induced resistance to alectinib via the inhibition of Gab1

**DOI:** 10.1038/s41419-020-2307-5

**Published:** 2020-02-10

**Authors:** Hengyi Chen, Caiyu Lin, Tao Peng, Cheng Hu, Conghua Lu, Li Li, Yubo Wang, Rui Han, Mingxia Feng, FenFen Sun, Yong He

**Affiliations:** 1 Department of Respiratory Disease, Daping Hospital, Army Medical University, Chongqing, 400042 China; 2 Department of Geriatric Medicine, Dazhou Central Hospital, Sichuan, 635000 China

**Keywords:** Drug development, Preclinical research

## Abstract

Alectinib is a second-generation anaplastic lymphoma kinase (ALK) inhibitor that has sufficient clinical efficacy and satisfactory safety in ALK-positive non-small cell lung cancer (NSCLC) patients with or without brain metastasis. Alectinib has now become an important drug in the first-line treatment of advanced ALK-positive NSCLC; however, resistance is almost inevitable. The increased expression of hepatocyte growth factor (HGF) and its physiological receptor tyrosine kinase MET have been shown to be linked to acquired resistance to various tyrosine kinase inhibitors (TKIs), and this phenomenon has been observed in some ALK-positive NSCLC tumour tissues. In this study, we found that HGF levels in the culture supernatant of an ALK-positive cell line tended to increase with time and could be further increased by alectinib in a time-dependent manner. Exogenous or endogenous HGF did not cause resistance to the ALK/MET double-targeted small molecule inhibitor crizotinib, but it was an important cause of alectinib resistance. Furthermore, Gab1 was a key effector in the HGF/MET signal transduction pathway that mediated alectinib resistance. The antidiabetic drug metformin combined with alectinib overcame alectinib resistance triggered by HGF/MET through disrupting the complex between MET and Gab1, thereby inhibiting Gab1 phosphorylation and the activation of downstream signal transduction pathways. These results suggest that metformin combined with alectinib may be useful for overcoming alectinib resistance induced by the activation of the HGF/MET signalling pathway and improving the efficacy of alectinib.

## Introduction

The first-generation anaplastic lymphoma kinase (ALK)-tyrosine kinase inhibitor (TKI) crizotinib has multiple kinase inhibitory activities against ALK, MET, and ROS1, and it shows promising efficacy for non-small-cell lung cancer (NSCLC) patients carrying the ALK gene rearrangement^1,2^. However, almost all patients who strongly responded to crizotinib eventually develop drug resistance within the first year of treatment^3,4^. Next-generation ALK inhibitors, including alectinib, have high selectivity for ALK, and they have demonstrated promising activity and satisfactory safety in both crizotinib-pretreated and crizotinib-naïve populations^5,6^. Furthermore, alectinib demonstrated superior CNS activity versus that of crizotinib in ALK-positive NSCLC patients^6^, and it has been approved for the first-line treatment of ALK-positive NSCLC patients. However, as with crizotinib, acquired resistance remains a limitation of its efficacy^7^.

The mechanisms of resistance to alectinib and crizotinib presumably differ because of the difference in the inhibition of MET by these two agents. Hepatocyte growth factor (HGF) and its physiological receptor tyrosine kinase MET have been reported to be involved in acquired resistance to various TKIs and have been seen as critical targets in cancer therapy^8,9^. Studies have found that the abnormal expression of HGF and MET are substantially more frequent in ALK-positive patients than in ALK-negative patients^10^, which may be an important cause of resistance to alectinib^11,12^ but not to crizotinib. Although a recent study showed that Hsp90 inhibitors may overcome ligand-triggered resistance to alectinib^13^, the involved mechanism is still unclear, and severe toxicity limits their clinical application. Metformin-based combinatorial therapy has been demonstrated as an effective method for improving the sensitivity of TKI-resistant cancer cells to TKIs^14^. Moreover, metformin treatment significantly improved the survival of lung cancer patients with epidermal growth factor receptor (EGFR)-activating mutations and type 2 diabetes^15^. We also showed that metformin restores crizotinib sensitivity in crizotinib-resistant NSCLC cells through the inhibition of the insulin-like growth factor 1 receptor (IGF1-R) signalling pathway^16^. It is worth noting that our previous study found that metformin exerted an inhibitory effect on downstream signalling mediators of MET, such as AKT and ERK^14^, which encouraged us to investigate whether metformin could restore alectinib sensitivity in alectinib-resistant cells with high expression of HGF and MET.

In the present study, we attempted to determine whether metformin could be used as a therapeutic agent for overcoming HGF-induced alectinib resistance and further explored the mechanism underlying HGF overexpression in NSCLC cells with an ALK rearrangement.

## Materials and methods

### Cell culture and reagents

The H3122 human lung adenocarcinoma cell line with EML4-ALK fusion protein variant 1 (E13; A20) was obtained from Shanghai Bioleaf Biotech Co., Ltd. (Shanghai, China). The H2228 human lung adenocarcinoma cell line with EML4-ALK fusion protein variant 3 (E6; A20) was obtained from American Type Culture Collection (Manassas, VA, USA). Cells with acquired resistance to alectinib were established by exposing parental cells to increasing concentrations of alectinib (20 nmol/L to 1 μmol/L) for 10 months and selecting clones using the limiting dilution method. Six clones with different MET levels were isolated from the resultant alectinib‐resistant H3122 cell lines (H3122-AR1-6). Human HGF gene-transfected cells (H3122/HGF and H2228/HGF) and vector control cells (H3122/Vec and H2228/Vec) were established as described^17^. Cells were cultured in Roswell Park Memorial Institute 1640 medium (RPMI-1640, HyClone) with Earle’s salts supplemented with 10% foetal bovine serum (FBS, Gibco), 2 mmol/L l-glutamine solution (Gibco), 100 U/mL penicillin (HyClone) and 100 µg/mL streptomycin (HyClone) in a humidified CO_2_ incubator at 37 °C.

Alectinib (ALK-selective inhibitor, S2762) and JNJ-38877605 (MET-selective inhibitor, S1114) were purchased from Selleck Chemicals (Houston, TX, USA). HGF (294-HG) was purchased from R&D Systems (Minneapolis, MN, USA). Metformin (Sigma, 1115-70-4) was dissolved in deionized water and stored at −20 °C. Compound C (an AMPK inhibitor, cat#171261) was purchased from Calbiochem.

### MTT assay

The growth inhibitory effects were examined using an MTT dye reduction assay. A total of 2000 tumour cells were plated in 100 µL of culture medium in 96-well microtiter plates and incubated in medium containing 10% FBS for 24 h. Alectinib, metformin, JNJ-38877605 and/or HGF were added to each well as indicated, and incubation continued for another 48 h. Ten microliters of 5 mg/mL MTT reagent (Sigma) was added to each well, followed by incubation for 4 h at 37 °C. Then, the medium was removed, and the formazan crystals in each well were dissolved in 150 µL of dimethyl sulfoxide (DMSO). The absorbance was measured with a ThermoFisher Spectrophotometer 1510 (Molecular Devices, Inc.) at a wavelength of 490 nm. The percentage growth was calculated relative to that of untreated controls. The experiments were conducted in triplicate.

### Colony-formation assay

For the colony-formation assay, cells were plated at 2000 cells per well, resuspended in RPMI and seeded and incubated in six-well plates for 14 days. Then, the cells were washed and fixed with 3.7% paraformaldehyde. Next, the colonies were fixed and stained with 0.1% crystal violet stain. Colonies with a diameter greater than 1 mm were counted. Triplicate samples were used in the experiment.

### Ki67-incorporation assay

Cell proliferation was also examined by a Ki67-incorporation assay using a Ki67 labelling and detection kit (Sigma). The cells were treated with alectinib, metformin, or HGF for 48 h and then incubated with Ki67 (1:200 dilution) and fixed for 6 h. The cell nuclei were counterstained with 4′,6-diamidino-2-phenylindole (DAPI) and viewed with a live cell station (Delta Vision, API). At least 500 cells from three independent experiments were counted. Triplicate samples were used in the experiment. The data were expressed as the mean value of the percentage of positive cells ± standard deviation (SD).

### ELISA

HGF levels were measured using a sandwich ELISA (R&D Systems). The cells were treated with alectinib or crizotinib for 12, 24, or 48 h. The supernatants were harvested and clarified by centrifugation prior to being diluted 1:10 in PBS/1% BSA and assayed according to the manufacturer’s instructions. Triplicate samples were used in the experiment.

### Transfection of siRNA

H3122 cells were transfected with negative control (100 pmol/L) or Gab1 siRNA (100 pmol/L) (Santa Cruz Biotechnology, Santa Cruz, CA) using the Oligofectamine reagent (Invitrogen, Grand Island, NY). The target sequences used for the siRNAs were as follows: si-h-Gab1#1 (5′-CCAAGAAGCCTATTCGTATdTdT-3′); si-h-Gab1#2 (5′-GCCTCAGACACTGACAGTA-3′); and si-h-Gab1#3 (5′-GCAGATGAGAGAGTGGATT-3′). The cells were harvested at 48 h posttransfection. Western blot analysis was performed to monitor Gab1 expression. The experiments were conducted in triplicate.

### Lentivirus production and transduction

The human Gab1 cDNA sequence (Genebank accession number: NM_002039) was searched for suitable target sequences. Lenti-p-Gab1^Y627^A (mutations in Gab1 that abolish overall phosphorylation of Tyr627, NM_002039(Y627A)), Lenti-p-Gab1^Y627^D (mutations in Gab1 that cause continuous phosphorylation of Tyr627, NM_002039(Y627D)), Lenti-wild-type p-Gab1 (NM_002039) and Lenti-NC virus were designed and generated by Shanghai GeneChem Co., Ltd. DNA oligos containing the target sequence were inserted into the GV492 vector by double digestion with BamHI and AgeI. H3122 cells were transfected with ViraPower packaging mix using Lipofectamine 2000 reagent according to manufacturer’s instructions.

### Western blot analysis

The cells, which were grown and treated as indicated, were collected. Whole cell lysates were prepared using EBC lysis buffer, which contained 50 mmol/L Tris–HCl [pH 8.0], 120 mmol/L NaCl, 1 mmol/L EDTA, 1 mmol/L EGTA, 0.3 mmol/L phenylmethylsulfonyl fluoride, 0.2 mmol/L sodium orthovanadate, 1% Triton X-100, 0.5% NP-40, and 5 U/mL aprotinin. The proteins were separated using SDS-PAGE and transferred to PVDF membranes (Invitrogen).

The following antibodies from Cell Signalling Technology were used: MET (8198S), Gab1 (3232S), p-Gab1 (Y627, 3233S), AKT (4685S), p-AKT (Ser473, 4060S), ERK (ERK1/2, 9102S), p-ERK (Thr202/Tyr204, 4370S), mTOR (2983S), p-mTOR (Ser2448, 5536S), P70S6K (2708P), p-P70S6K (Thr389, 9234S), S6 (#2217S), p-S6 (Ser240/244, 4858S), AMPK (2603S), and ALK (3333S). HGF (ab178395) and p-MET (Y1349, ab68141) were purchased from Abcam Technology. P-AMPK (Thr172, A5194) and GAPDH (A5028) were purchased from Selleck Technology.

### Tumour xenograft models

The H3122/HGF and H3122 cell xenografts were generated as previously described^14^. For each cell type, twenty-four female 6-week-old BALB/cA-nu mice (Laboratory Animal Centre of the Army Medical University, Chongqing, China) were randomly divided into four groups with allocation concealment. Subsequently, a total of 2 × 10^6^ H3122/HGF or H3122 cells were injected subcutaneously into the back next to the left forelimb. The tumour volumes were estimated according to (length × width^2^)/2 and measured twice weekly. When the tumours reached 30 mm^3^, the mice were treated every day orally with placebo, metformin (1 mg/mL), alectinib (0.02 mg/mL), or both metformin and alectinib. Animal caretakers and investigators conducting the experiments were blinded to the allocation sequence. Investigators assessing, measuring and quantifying experimental outcomes were blinded to the intervention. All animals that were entered into this experiment actually completed it, and no data were removed before analysis. The tumour volume estimation at the end of the experiment was performed as previously described^14^. All animal experiments in the study were performed in alignment with the recommendations in the Guide for the Care and Use of Laboratory Animals. All animal protocols were approved by the Ethics Committee of the Army Medical University.

### Statistical analysis

All data are presented as the mean ± SD. Statistical analyses were performed using unpaired, two-tailed Student’s *t*-tests. A *p*-value of <0.05 was considered significant. Statistical analyses were conducted using SPSS 17.0 software.

## Results

### Activation of the HGF/MET signalling pathway is an important cause of resistance to alectinib but not to crizotinib

**Fig. 1:**
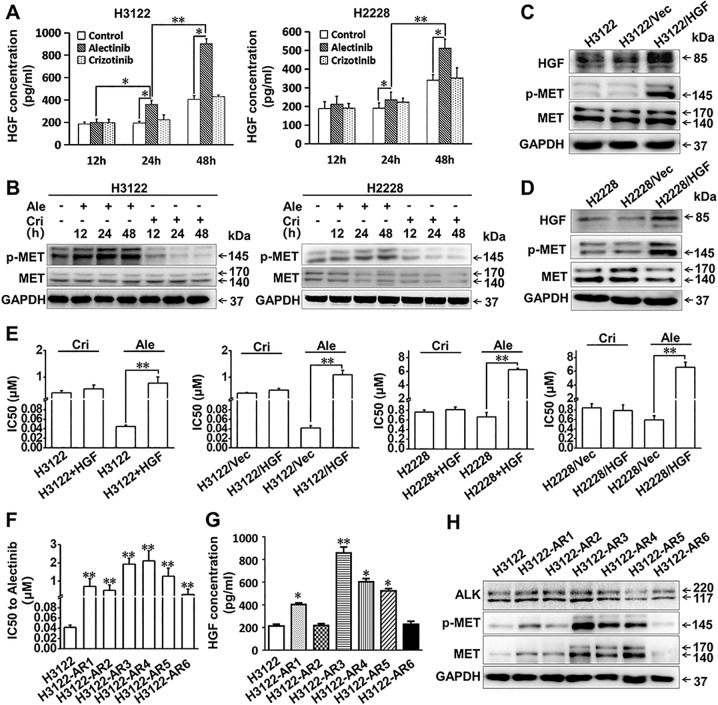
The HGF/MET signalling pathway contributes to resistance to alectinib in ALK-positive lung cancer cells. **a** The concentrations of HGF in the H3122 and H2228 cell culture supernatants with or without alectinib (50 nmol/L) or crizotinib (100 nmol/L) administration were determined by ELISA. The data are presented as the mean ± SD, and the experiment was repeated three times. *p *<* 0.05; **p *<* 0.001. **b** H3122 and H2228 cells were treated with alectinib (50 nmol/L) or crizotinib (100 nmol/L) for the indicated amounts of time, and MET phosphorylation was measured by western blotting. **c**, **d** H3122 and H2228 cells were transfected with HGF, and the expression of HGF and the phosphorylation levels of MET were determined by western blotting. Vec negative control vector. **e** Cell proliferation in crizotinib-treated and alectinib-treated cells with or without HGF was examined by MTT assay; the IC50 values for crizotinib and alectinib were calculated. The data are presented as the mean ± SD, and the experiment was repeated three times. **p *<* 0.001. Cri crizotinib, Ale alectinib, Vec negative control vector. **f** H3122-AR1-6 cells are resistant to alectinib. The alectinib IC50 values of H3122 and H3122-AR cells were detected by MTT assay. The data are presented as the mean ± SD from three independent experiments. **p *<* 0.001 compared with H3122 cells. **g** Detection of HGF in the cell culture supernatants of H3122 and H3122-AR cells by ELISA. The data are presented as the mean ± SD from three independent experiments. Asterisks (*) indicates *p* *<* 0.05 compared with parental H3122 cells; **p *<* 0.001 compared with parental H3122 cells. **h** Immunoblotting analysis of ALK, MET, and the phosphorylation of MET in H3122 and H3122-AR cell lines.

Previous studies have found evidence that MET receptor expression is significantly increased in ALK-positive NSCLC^10^ and that the tumour microenvironment is altered during TKI treatment, which may increase the production of HGF and contribute to resistance onset^18^. We then performed an ELISA assay to determine HGF levels in cultured media obtained from H3122 and H2228 cells. The results showed that HGF levels continuously increased for 48 h. Additionally, crizotinib had no obvious effect on HGF levels, but alectinib treatment led to large increases in HGF production in H3122 and H2228 cells in a time-dependent manner (Fig. 1a). Moreover, western blot analyses demonstrated that crizotinib inhibited the phosphorylation of MET. In contrast to crizotinib, alectinib treatment did not inhibit MET phosphorylation but instead increased MET phosphorylation at the measured time points (12, 24, or 48 h) (Fig. 1b). These results suggest that HGF/MET signalling is activated after treatment with alectinib.

Next, we investigated the effect of increased levels of HGF on alectinib sensitivity. We induced HGF overexpression with recombinant lentiviral transfection in vitro. The protein expression levels of HGF in H3122/HGF and H2228/HGF were significantly upregulated, suggesting that lentiviral infection of the H3122 and H2228 cells was effective. Further, the levels of MET phosphorylation were markedly elevated (Fig. 1c, d). There was no difference in the morphology of the primary cells and the transfected cells according to microscopic observation. Furthermore, the sensitivities of the H3122 and H2228 cells to alectinib or crizotinib were evaluated using an MTT assay. The MTT results showed that H3122 and H2228 cells were resistant to alectinib when transfected with HGF or in the presence of exogenous HGF (50 ng/mL), whereas the cells were sensitive to crizotinib in the presence of HGF (Table 1; Fig. 1e).

**Table 1:** IC50 values of alectinib and crizotinib.

	H3122 (IC50 µmol/L)	H2228 (IC50 µmol/L)
Alectinib		
HGF (−)	0.046 ± 0.014	0.665 ± 0.090
HGF (50 ng/mL)	0.775 ± 0.230	6.233 ± 0.210
/Vec	0.042 ± 0.005	0.594 ± 0.089
/HGF	1.099 ± 0.162	6.577 ± 0.791
Crizotinib		
HGF (−)	0.409 ± 0.079	0.765 ± 0.041
HGF (50 ng/mL)	0.552 ± 0.150	0.811 ± 0.060
/Vec	0.418 ± 0.002	0.841 ± 0.089
/HGF	0.513 ± 0.068	0.784 ± 0.125

*IC50* 50% inhibitory concentration.

**Fig. 2:**
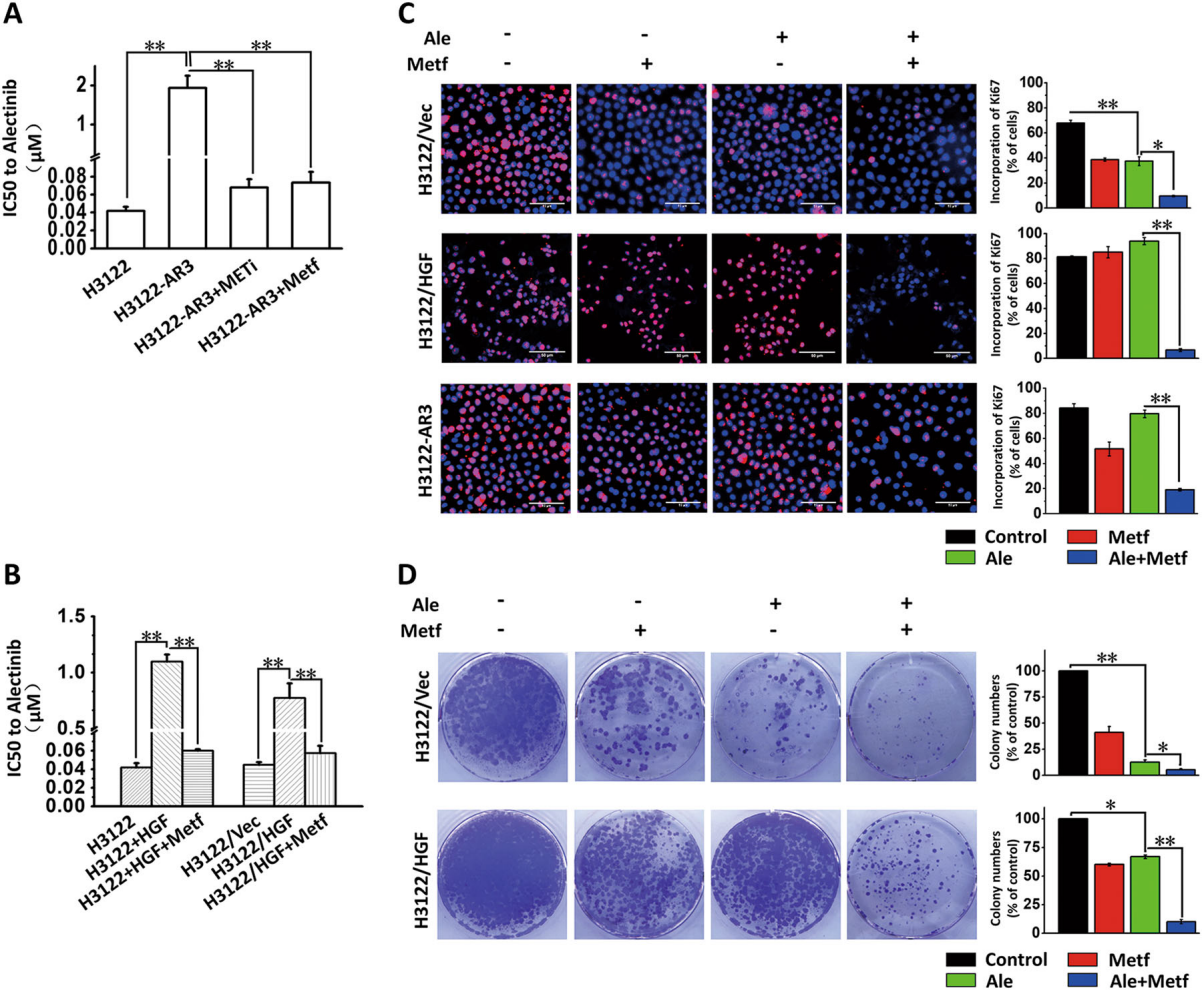
Metformin reversed the alectinib resistance induced by HGF/MET in vitro. **a** The alectinib IC50 values of H3122-AR3 cells with or without JNJ-38877605 (10 nmol/L) or metformin (5 mmol/L) treatment. **p *<* 0.001. **b** Metformin reversed alectinib resistance induced by exogenous or endogenous HGF in H3122 cells. The alectinib IC50 values of H3122 cells, H3122/Vec cells, and H3122/HGF cells with or without HGF (50 ng/mL) and metformin (5 mmol/L) treatment were detected by MTT assay. The data are presented as the mean ± SD from three independent experiments. **p *<* 0.001. **c** Metformin (5 mmol/L) and alectinib (50 nmol/L) synergistically inhibited the proliferation of H3122/Vec cells, H3122/HGF cells and H3122-AR3 cells, as determined by a Ki67-incorporation assay. The data are presented as the mean ± SD from three independent experiments. *p *<* 0.05; **p *<* 0.001. **d** Metformin (5 mmol/L) and alectinib (50 nmol/L) synergistically inhibited the clone-forming ability of H3122/Vec cells and H3122/HGF cells. *p *<* 0.05; **p *<* 0.001. Ale alectinib, METi MET selective inhibitor JNJ-38877605, Metf metformin, Vec negative control vector.

The H3122 cells were grown initially in medium containing 20 nmol/L alectinib, and the concentration was gradually increased to 1 μmol/L over the subsequent 10 months. Then, monoclonal cell lines were selected, and six alectinib-resistant monoclonal cell lines (H3122-AR1, H3122-AR2, H3122-AR3, H3122-AR4, H3122-AR5, and H3122-AR6) were obtained (Fig. 1f). The HGF levels in H3122-AR3, H3122-AR4, and H3122-AR5 cells culture media were significantly increased (Fig. 1g). Furthermore, the expression and phosphorylation levels of MET in above three cells were significantly higher than those in the parental cells (Fig. 1h). The MTT assay showed that cotreatment of the MET-selective inhibitor JNJ-38877605 (10 nmol/L) restored sensitivity to alectinib in H3122-AR3 cells (Fig. 2a). Overall, these data suggested that elevated levels of HGF production and MET activation were important contributors to alectinib resistance.

### Metformin reverses the alectinib resistance induced by HGF/MET in ALK-positive NSCLC cells

We next tested the hypothesis that metformin may restore alectinib sensitivity in H3122-AR3 cells. As shown in Fig. 2a, metformin treatment (5 mmol/L) restored sensitivity to alectinib in H3122-AR3 cells to the level observed in the parental H3122 cells. Furthermore, because the stimulation of H3122 cells by exogenous HGF or HGF overexpression could lead directly to alectinib resistance, we further determined whether metformin could overcome the alectinib resistance induced by HGF. The MTT results indicated that the addition of metformin (5 mmol/L) reversed the alectinib resistance induced by HGF (Fig. 2b). According to the results of our previous study^16^, the in vitro dose of metformin used in this study was 5 mmol/L, which has minimal influence on ALK-positive NSCLC cell growth. Next, we performed the Ki67-incorporation assay and/or the colony-forming assay in H3122/Vec, H3122/HGF, and H3122-AR3 cells. The results showed that metformin or alectinib alone slightly decreased the proliferation of H3122/HGF and H3122-AR3 cells and the colony formation of H3122/HGF cells, whereas the combination of the drugs significantly enhanced the inhibitory effect (Fig. 2c, d). Overall, these data suggest that metformin could overcome the alectinib resistance induced by the HGF/MET signalling pathway.

### Metformin in combination with alectinib does not inhibit MET activation but significantly inhibits the downstream signalling of HGF/MET

**Fig. 3:**
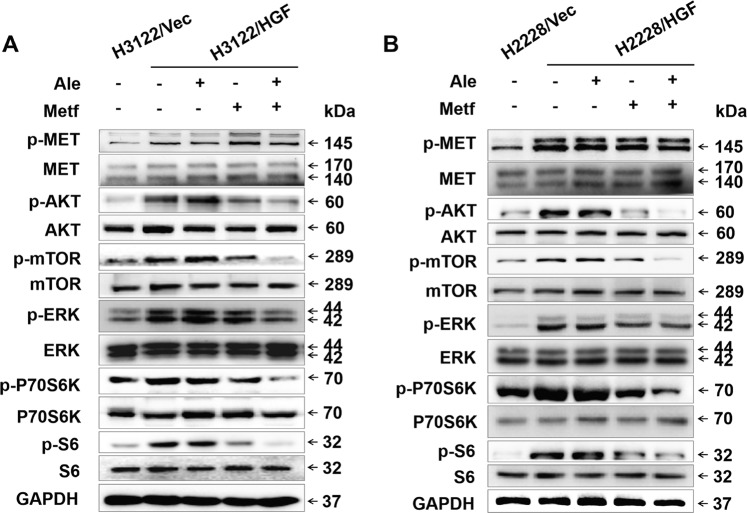
Metformin decreased HGF-induced AKT/mTOR/P70S6K and ERK pathway activation. **a** H3122/Vec and H3122/HGF cells were lysed after treatment with or without alectinib (50 nmol/L) and/or metformin (5 mmol/L) for 48 h and were subjected to western blot analysis for the indicated proteins. **b** H2228/Vec and H2228/HGF cells were lysed for western blotting after treatment with or without alectinib (500 nmol/L) and/or metformin (5 mmol/L) for 48 h. GAPDH was used as an internal control. Similar results were obtained in three independent experiments. Ale alectinib, Metf metformin, Vec negative control vector.

To identify the molecular mechanisms of metformin involved in overcoming acquired resistance to alectinib triggered by HGF, the present study aimed to detect the signalling molecules downstream of HGF/MET following HGF over expression in H3122 and H2228 cells. As shown in Fig. 3a, b, western blot analysis indicated that endogenous HGF remarkably increased the phosphorylation of MET and downstream AKT, mTOR, ERK, P70S6K, and S6, which were not inhibited by alectinib. Metformin in combination with alectinib significantly reduced the phosphorylation levels of AKT, mTOR, ERK, P70S6K, and S6 induced by HGF. However, alectinib alone or combined with metformin did not inhibit the HGF-induced phosphorylation of MET (Fig. 3a, b). These results suggest that metformin overcame alectinib resistance triggered by HGF mainly via inhibiting the signalling transduction pathways downstream of HGF/MET.

### The reversal of alectinib resistance by metformin does not depend on AMPK activation

**Fig. 4:**
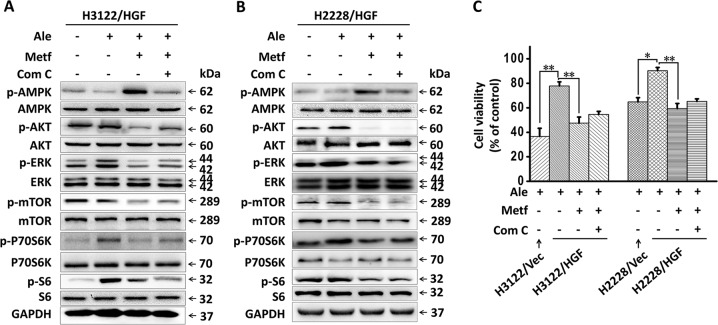
The inhibitory effect of metformin on AKT/mTOR/P70S6K and ERK signalling pathways was independent of AMPK activation. **a**, **b** Western blot analyses of H3122/HGF and H2228/HGF cells. The cells were treated with alectinib (100 nmol/L), metformin (5 mmol/L) and/or compound C (1 µmol/L) for 48 h. Cell lysates were harvested and subjected to western blot analysis for the indicated proteins. GAPDH was used as an internal control. Similar results were obtained in three independent experiments. **c** Metformin (5 mmol/L) and alectinib (100 nmol/L) synergistically inhibited the proliferation of H3122/HGF and H2228/HGF cells, and this effect was hardly affected by compound C (1 µmol/L). The data are presented as the mean ± SD from three independent experiments. *p *<* 0.05; **p *<* 0.001. Ale alectinib, Metf metformin, Com C AMPK selective inhibitor compound C, Vec negative control vector.

Metformin is known to activate AMPK in various tissues^19,20^. We used western blot analysis to confirm whether metformin inhibited the signalling transduction pathways downstream of HGF/MET and overcame HGF-induced alectinib resistance via activating AMPK. As shown in Fig. 4a, b, metformin effectively increased the phosphorylation of AMPK in the presence of alectinib in H3122/HGF and H2228/HGF cells. Next, we investigated whether the effect of metformin depended on AMPK activation. Compound C, an AMPK-specific inhibitor, was used to inhibit the activation of AMPK by metformin. The results showed that treatment with compound C (1 µmol/L) reversed metformin-induced AMPK phosphorylation but could not restore the phosphorylation levels of AKT, ERK, mTOR, P70S6K, and S6, which were inhibited by metformin in H3122/HGF and H2228/HGF cells (Fig. 4a, b). In accordance with this finding, the MTT results indicated that compound C (1 µmol/L) did not significantly reduce the effect of metformin on overcoming HGF-induced alectinib resistance in both H3122/HGF and H2228/HGF cells (Fig. 4c). These results suggest that the effect of metformin on inhibiting HGF/MET downstream signalling pathways and overcoming HGF-induced alectinib resistance did not depend on AMPK activation.

### Metformin inhibited Gab1, a key effector in the HGF/MET signal transduction pathway, while reversing the alectinib resistance induced by HGF

**Fig. 5:**
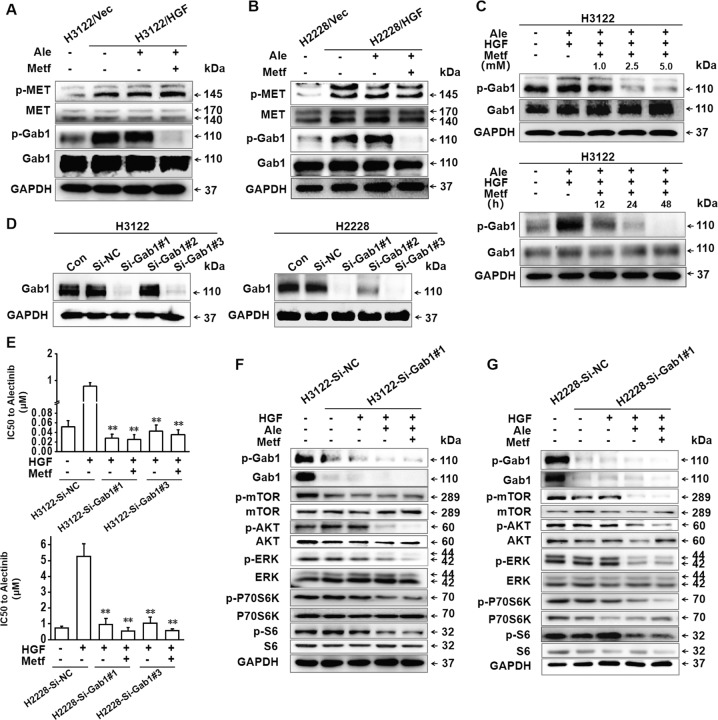
Metformin reversed HGF-induced alectinib resistance via inhibiting Gab1 activity. **a** H3122/Vec cells, H3122/HGF cells and H3122/HGF cells pretreated with alectinib (50 nmol/L) and/or metformin (5 mmol/L) for 48 h were lysed and subjected to western blot analysis to evaluate the phosphorylation of MET, MET, phosphorylation of Gab1 and Gab1. GAPDH was used as an internal control. Similar results were obtained in three independent experiments. **b** H2228/Vec cells, H2228/HGF cells and H2228/HGF cells pretreated with alectinib (500 nmol/L) and/or metformin (5 mmol/L) for 48 h were lysed and subjected to western blot analysis for phosphorylation of MET, MET, phosphorylation of Gab1 and Gab1. GAPDH was used as an internal control. Similar results were obtained in three independent experiments. **c** H3122 cells were treated with 50 nmol/L alectinib, 50 ng/mL HGF and metformin with the indicated doses (48 h) and for the indicated times (5 mmol/L), and the phosphorylation levels of Gab1 were assessed by western blotting. Similar results were obtained in three independent experiments. **d** Gab1-specific siRNAs were introduced into H3122 and H2228 cells. After incubation for 48 h, the cells were lysed, and Gab1 protein was detected by western blot analysis. **e** Negative control, Gab1-specific siRNA#1 or Gab1-specific siRNA#3 were introduced into H3122 and H2228 cells. The indicated cells (control, treated with 50 ng/mL HGF or treated with 50 ng/mL HGF plus 5 mmol/L metformin) were incubated with the indicated doses of alectinib for 48 h, and the IC_50_ values of indicated cells for alectinib were detected by MTT assay. The data are presented as the mean ± SD from three independent experiments. **p *<* 0.001 compared with homologous Si-NC cells treated with HGF (50 ng/mL). **f**, **g** The indicated cells were incubated with HGF (50 ng/mL), with or without alectinib (50 nmol/L for H3122-Si-NC and H3122-SiGab1#1, 500 nmol/L for H2228-Si-NC and H2228-SiGab1#1) and metformin (5 mmol/L) for 48 h. The cells were then lysed, and the indicated proteins were detected by western blot analysis. Similar results were obtained in three independent experiments. Ale alectinib, Metf metformin, Con control, NC negative control.

According to previous studies^21^, Gab1 is a key effector of the activation of MET by HGF; MET typically signals in tandem with Gab1, the activation of MET by phosphorylation promotes the recruitment and tyrosine phosphorylation of Gab1. The Gab1/MET module in turn recruits multiple proteins and mediates the downstream signalling molecules of HGF/MET, leading to cell survival, motility, and morphogenesis^21,22^. To further explore the molecular mechanisms of metformin involved in overcoming HGF-induced alectinib resistance, western blot analysis was conducted to investigate the expression and activation of Gab1 in H3122 and H2228 cells with or without HGF overexpression. Compared with that in H3122/Vec and H2228/Vec cells, the phosphorylation of MET and Gab1 were obviously increased in HGF-overexpressing H3122/HGF and H2228/HGF cells, and this effect could not be inhibited by alectinib. Metformin in combination with alectinib substantially decreased Gab1 phosphorylation but had little influence on the phosphorylation level of MET (Fig. 5a, b). In addition, as shown in Fig. 5c, metformin decreased the phosphorylation levels of Gab1 in a dose-dependent and time-dependent manner in the presence of exogenous HGF (50 ng/mL). We next suppressed endogenous Gab1 expression in H3122 and H2228 cell lines using the transfection of Gab1-specific siRNA. The efficacy of Gab1 knockdown was confirmed by western blot analysis (Fig. 5d). MTT results showed that the siRNA-mediated knockdown of Gab1 dramatically restored the sensitivity to alectinib of H3122 and H2228 cells even if exogenous HGF (50 ng/mL) was present; there was no obvious change in alectinib sensitivity with the addition of metformin (Fig. 5e). After Gab1 was knocked down, western blot analysis found that the phosphorylation levels of mTOR, AKT, ERK, P70S6K, and S6 could not be increased by HGF but could be significantly reduced by alectinib even in the presence of exogenous HGF (50 ng/mL). Moreover, additional metformin could not further inhibit the phosphorylation of these proteins (Fig. 5f, g).

### Metformin inhibited Gab1 phosphorylation via blocking the interaction of MET and Gab1

**Fig. 6:**
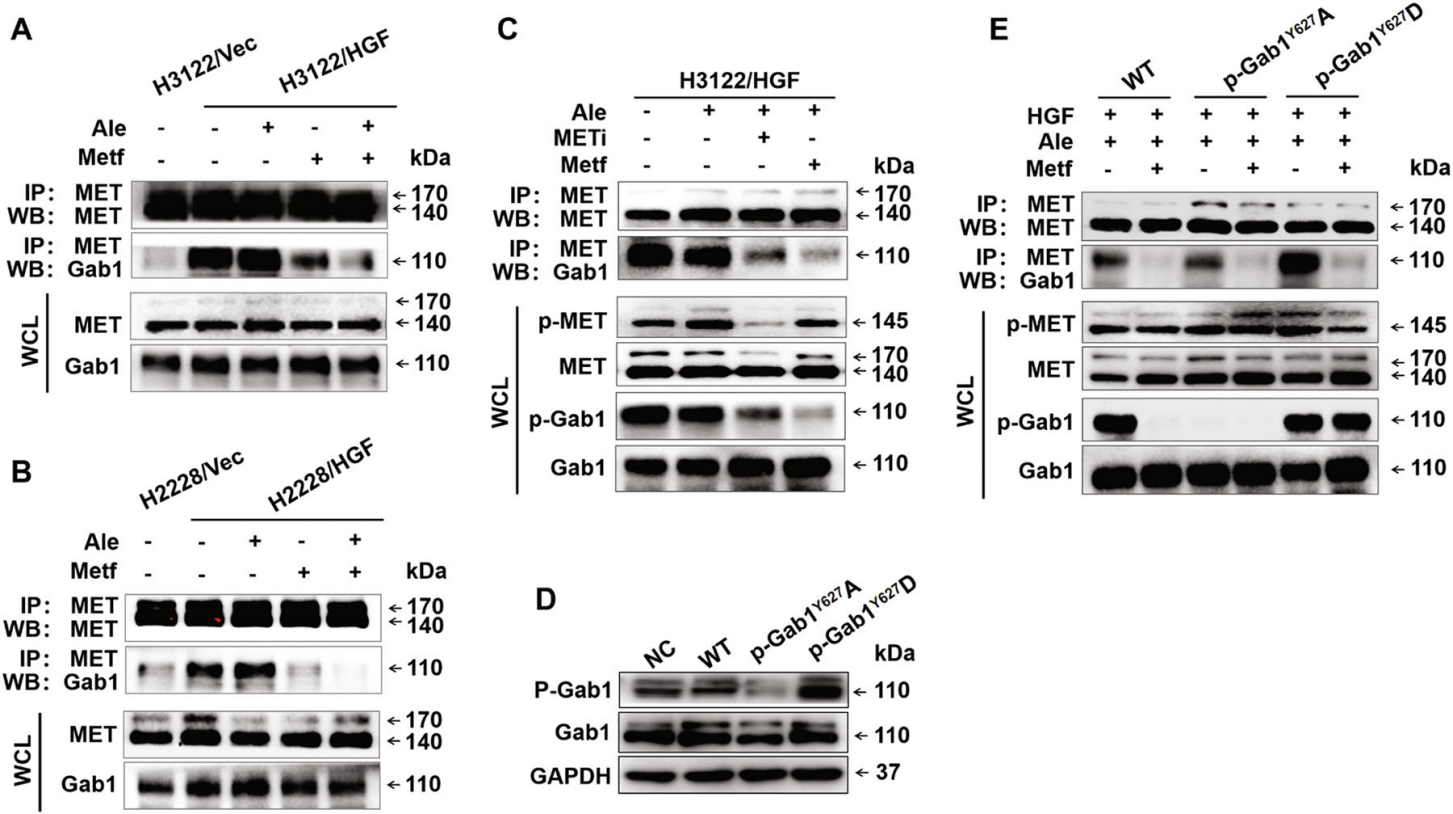
Metformin blocked the interaction of MET and Gab1 and thereby inhibited the phosphorylation of Gab1. **a** H3122/Vec and H3122/HGF cells pretreated with alectinib (50 nmol/L) and/or metformin (5 mmol/L) for 48 h were lysed and then subjected to denaturing Co-IP with M2 beads followed by western blot analysis. Similar results were obtained in three independent experiments. **b** H2228/Vec and H2228/HGF cells pretreated with alectinib (500 nmol/L) and/or metformin (5 mmol/L) for 48 h were lysed and then subjected to denaturing Co-IP with M2 beads followed by western blot analysis. Similar results were obtained in three independent experiments. **c** H3122/HGF cells were treated with alectinib (50 nmol/L), JNJ-38877605 (10 nmol/L) and/or metformin (5 mmol/L) for 48 h. Cell lysates were harvested and subjected to denaturing Co-IP with M2 beads followed by western blot analysis. Similar results were obtained in three independent experiments. **d** H3122 cells were transfected with p-Gab1^Y627^A-lentivirus, p-Gab1^Y627^D-lentivirus, wild-type p-Gab1-lentivirus, and corresponding empty vector, and the phosphorylation levels of Gab1 were detected by western blot analysis. **e** H3122 cells were transfected with p-Gab1^Y627^A-lentivirus, p-Gab1^Y627^D-lentivirus, and wild-type p-Gab1-lentivirus and then treated with HGF (50 ng/mL) and alectinib (50 nmol/L) with or without metformin (5 mmol/L) for 48 h. Cell lysates were harvested and subjected to denaturing Co-IP with M2 beads followed by western blot analysis. Similar results were obtained in three independent experiments. Ale alectinib, Metf metformin, METi MET selective inhibitor JNJ-38877605, Vec negative control vector, NC empty vector, WT wild-type p-Gab1.

To characterise the specific mechanism involved in the inhibitory effect of metformin on Gab1, the interaction between MET and Gab1 was assessed by coimmunoprecipitation. Metformin treatment significantly disrupted the MET-Gab1 complex, as demonstrated by a remarkable reduction in their coimmunoprecipitation in the presence of metformin (Fig. 6a, b). The results also demonstrated that the MET-selective inhibitor JNJ-38877605 significantly inhibited the binding of Gab1 to MET while inhibiting MET phosphorylation and significantly inhibited Gab1 phosphorylation (Fig. 6c). This finding indicated that the phosphorylation status of MET affected the interaction between MET and Gab1. To clarify the effects of phosphorylation status of Gab1 on the interaction between MET and Gab1, H3122 cells were transfected with p-Gab1^Y627^A, p-Gab1^Y627^D, wild-type p-Gab1, and the corresponding empty vector (Fig. 6d). Co-immunoprecipitation experiments confirmed that metformin significantly reduced the interaction between MET and Gab1 regardless of whether Gab1 was phosphorylated (Fig. 6e). These findings suggest that metformin inhibited Gab1 phosphorylation via interfering with the sequestration of Gab1 by MET.

### Metformin plus alectinib potentiates alectinib-induced antitumour activity in alectinib-resistant and alectinib-sensitive mouse xenografts

To verify the in vivo effect of metformin on overcoming alectinib resistance induced by HGF or metformin on enhancing the inhibitory effect of alectinib in alectinib-sensitive tumours, H3122/HGF and H3122 cell xenograft models in nude mice were established. H3122/HGF and H3122 xenografts were treated with placebo, metformin, alectinib, and a combination for 4 weeks. According to a report by Iliopoulos^23^, the local tissue concentration of metformin is 1–10 mmol/L, which is several-fold higher than that in blood; therefore, the dose of metformin used in the in vivo experiments (1 mg/mL) was within the therapeutic range in humans, as described in our previous study^14^.

**Fig. 7:**
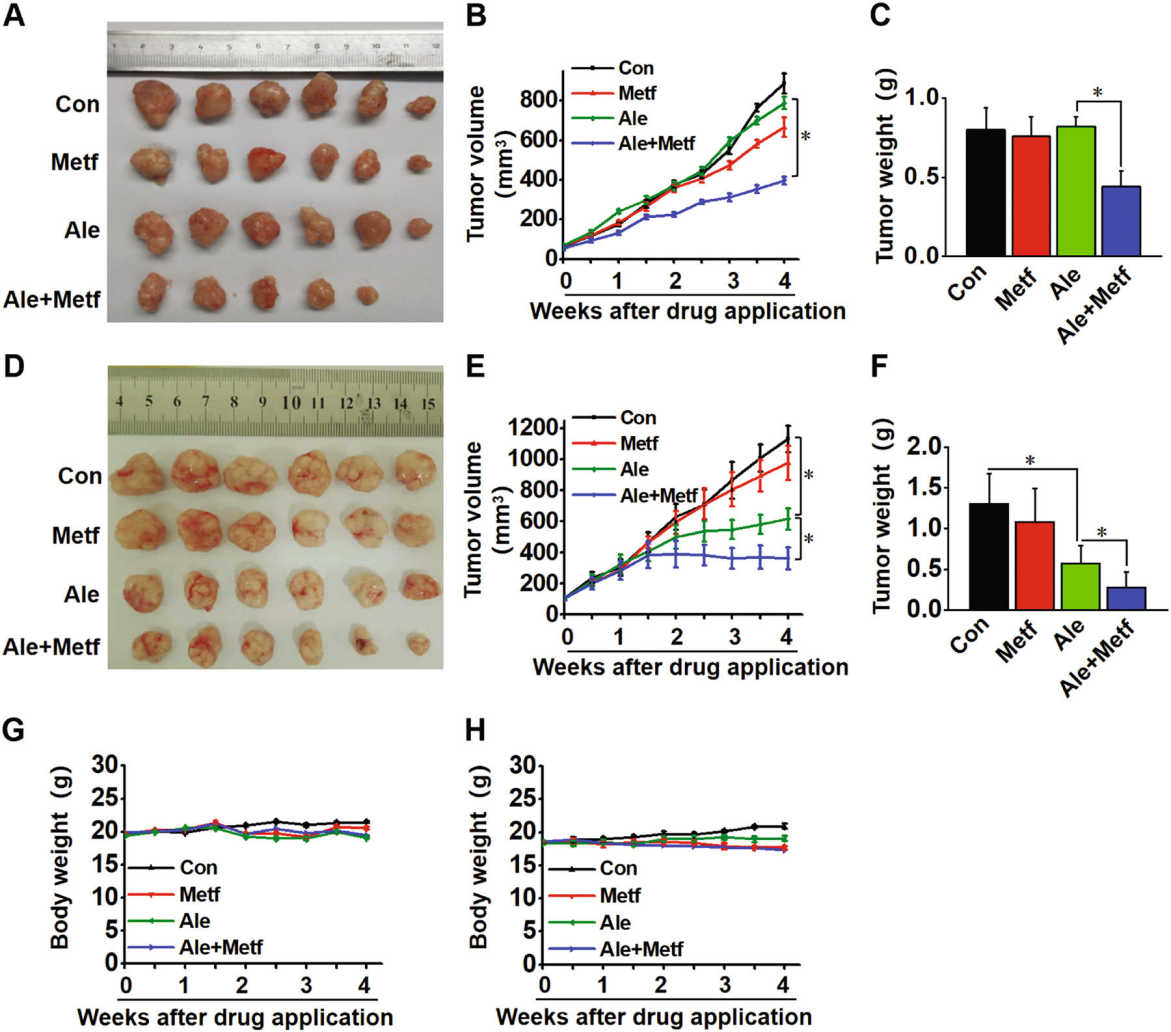
Efficacy of metformin combined with alectinib in the H3122/HGF and H3122 tumour xenograft models. **a** Placebo, alectinib (0.02 mg/mL), metformin (1 mg/mL), and alectinib (0.02 mg/mL) plus metformin (1 mg/mL) were administered by oral once daily to mice bearing. **a** H3122/HGF and **d** H3122 cell xenografts. Macroscopic appearance of the tumours at 4 weeks after drug administration. **b**, **c** Tumour volumes and tumour weights of H3122/HGF cell xenografts were measured twice a week. The data are the mean ± SE. *p *<* 0.05 (repeated measures model). **e**, **f** Tumour volumes and tumour weights of H3122 cell xenografts were measured twice a week. The data are the mean ± SE. *p *<* 0.05 (repeated measures model). The body weights of **g** H3122/HGF and **h** H3122 cell xenografts were measured twice a week. Con control, Metf metformin, Ale alectinib.

There were no significant differences in the volume and weight of tumours between mice treated with placebo, metformin alone or alectinib alone in H3122/HGF xenografts (Fig. 7a–c). In contrast to H3122/HGF xenografts, the growth of H3122 xenografts were effectively inhibited by alectinib alone (Fig. 7d–f). However, regardless of H3122/HGF and H3122 xenografts, metformin in combination with alectinib produced a significantly greater inhibition of tumour growth compared with that in the single agent groups (*p* < 0.05). The tumour in one case of an H3122/HGF xenograft treated with both metformin and alectinib disappeared in the fourth week. Furthermore, no appreciable weight loss was observed in mice treated with placebo, metformin, alectinib, or both, suggesting that the combination regimen did not cause additional toxicity (Fig. 7g, h).

## Discussion

In this study, we found that there was a tendency towards an increase in the production of HGF in ALK-positive NSCLC cells over time; in particular, in the presence of alectinib, HGF production could be further increased. More importantly, the activation of the HGF/MET signalling pathway was shown to be an important factor leading to alectinib resistance. Metformin in combination with alectinib could overcome the alectinib resistance triggered by HGF via disrupting the MET-Gab1 complex and then inhibiting the phosphorylation of Gab1, a key downstream effector of HGF/MET. Accordingly, metformin may be an ideal agent for overcoming HGF-triggered alectinib resistance in NSCLC with EML4-ALK rearrangement.

Drug resistance remains a vexing problem in the treatment of cancer patients. The aberrant activation of HGF and its receptor MET has been strongly implicated in the malignant transformation and progression of several tumours^8,24^ and is frequently implicated in resistance to targeted therapies^25,26^. Feng et al. found that significantly greater frequencies of high HGF and MET receptor expression were observed in ALK-positive NSCLC patients^10^. Furthermore, alectinib activates MET signalling even in the absence of HGF in a time-dependent manner^12^. Our experiments showed that alectinib induced a further increase in the production of HGF and the activation of MET over a longer period of time, and this effect was different from the effects of crizotinib, which could inhibit MET phosphorylation and had no effect on HGF production. Therefore, because of the absence of MET inhibitory activity, alectinib resistance may be associated with HGF/MET signalling. As expected, further research found that abnormal HGF expression triggers resistance to alectinib NSCLC cell with an ALK rearrangement^13^. Our findings confirmed these observations and indicated that HGF and MET expression levels were pertinent to the efficacy of alectinib. Furthermore, MET overexpression and HGF hypersecretion were detected in monoclonal H3122-AR cells. It is worth emphasising that abnormally activated HGF/MET signalling is probably an important cause of alectinib resistance, and its effects are completely the opposite of the effects of crizotinib. Based on these findings, the levels of HGF and the status of MET need to be taken into consideration before making therapeutic decisions and during therapy with alectinib.

A preclinical study has shown that the Hsp90 inhibitor 17-DMAG can enhance the efficacy of alectinib or overcome the alectinib resistance triggered by HGF^13^; however, high costs and severe side effects limit its clinical application^27,28^. Metformin is one of the most useful blood glucose-lowering drugs in clinical use and is of increasing interest due to its anticancer effects^29^. Metformin users among patients with diabetes mellitus were found to have a significantly lower risk of various types of tumours^30,31^, and metformin shows potent inhibition of a variety of tumour cells in vitro^32,33^. We previously reported that metformin, given in combination with EGFR-TKIs, shows significantly improved clinical efficacy in patients with NSCLC and type 2 diabetes mellitus^15^. Metformin overcomes IL-6-induced EGFR-TKI resistance in TKI-sensitive lung cancer cells through the inhibition of STAT3 and AKT phosphorylation and the enhancement of AMPK activation^14^. These findings indicated that metformin may delay the emergence of resistance to EGFR-TKIs in NSCLC patients. In the current study, we provided evidence that metformin in combination with alectinib dramatically reversed HGF-induced alectinib resistance in vitro and in vivo. Furthermore, metformin also dramatically reversed alectinib resistance in H3122-AR cells with high MET expression or HGF hypersecretion and significantly enhanced the sensitivity of alectinib in xenograft models in both H3122/HGF and H3122 cells. The results enriched the understanding of the use of metformin in TKI resistance. The use of alectinib combined with metformin may be a simple and clinically viable strategy for delaying and overcoming alectinib resistance.

Researchers have previously showed that HGF reduces susceptibility to alectinib by mainly restoring the downstream AKT and ERK pathways via MET activation, and the AKT-mTOR axis has important roles in the HGF/MET pathway^12,34^. This research found that metformin selectively inhibited the abnormal phosphorylation of AKT, mTOR, ERK, P70S6K, and S6 in the presence of HGF but had no effect on the phosphorylation of MET, regardless of the absence or presence of alectinib. These findings suggest that metformin reverses alectinib resistance induced by HGF/MET through the inhibition of the downstream signalling molecules of HGF/MET signalling pathway. It is necessary to further explore the intimate molecular mechanisms. Further study showed that even though AMPK activation is considered an important mechanism of the effects of metformin on auxiliary anticancer^35^, and metformin significantly increased the phosphorylation level of AMPK in this study, AMPK inhibition did not greatly influence the effect of metformin on inhibiting the AKT/mTOR/P70S6K and ERK signalling pathways or on overcoming alectinib resistance. Thus, we conclude that the reversal of alectinib resistance by metformin was not dependent on AMPK activation.

**Fig. 8:**
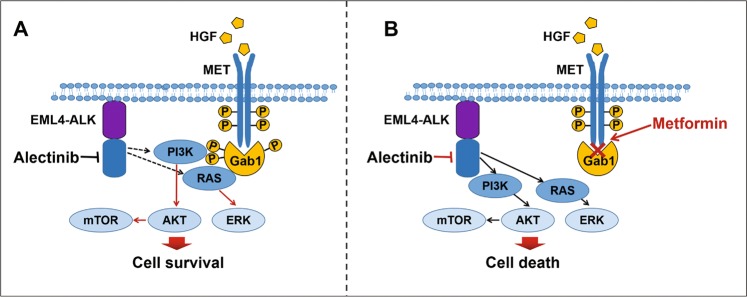
Diagram for the proposed effects of metformin in reducing alectinib resistance mediated by the HGF/MET signalling pathway. HGF induces alectinib resistance by activating the MET/Gab1 axis. HGF stimulates MET phosphorylation, and then promotes the integration of MET with Gab1, which is the main way of Gab1 phosphorylation; Gab1 phophorylating activates the AKT/mTOR and ERK signalling pathways and contributing to cell survival. Metformin blocks the binding of MET and Gab1 and then inhibits Gab1 phosphorylation and the activation of downstream signalling, leading to cell death.

Remarkably, Gab1 is an important signal effector in HGF/MET signalling pathway^21,36^. MET phosphorylation stimulated by HGF promotes the binding of Gab1 and MET, which is the main way of Gab1 phosphorylation, and then activates the AKT/mTOR and ERK signalling pathways downstream of MET^37^. Our results demonstrated that the MET-selective inhibitor JNJ-38877605 significantly inhibited the binding of Gab1 to MET, while inhibiting MET phosphorylation, and then significantly inhibited Gab1 phosphorylation. The phosphorylation status of Gab1 was mediated by the interactions between MET and Gab1. Interestingly, we found that siRNA-mediated knockdown of Gab1 dramatically restored alectinib sensitivity in the presence of HGF, which could not be further enhanced by metformin. More importantly, metformin could decrease Gab1 phosphorylation in a dose-dependent and time-dependent manner and disrupted the complex between MET and Gab1 regardless of Gab1 phosphorylation status. These results showed that the mechanism by which metformin overcame alectinib resistance was through disrupting the complex between MET and Gab1 and inhibiting Gab1 activation, thereby inhibiting downstream signal transduction pathways (Fig. 8).

In conclusion, the expression levels of HGF/MET play an important role in determining the efficacy of alectinib among patients with ALK-positive NSCLC. Metformin might be a potentially effective therapeutic tool for overcoming alectinib resistance associated with the HGF/MET signalling pathway.
